# Hemodynamic Comparison of Treatment Strategies for Intracranial Vertebral Artery Fusiform Aneurysms

**DOI:** 10.3389/fneur.2022.927135

**Published:** 2022-07-06

**Authors:** Yeqing Jiang, Gang Lu, Liang Ge, Rong Zou, Gaohui Li, Hailin Wan, Xiaochang Leng, Jianping Xiang, Xiaolong Zhang

**Affiliations:** ^1^Huashan Hospital, Fudan University, Shanghai, China; ^2^ArteryFlow Technology Co., Ltd, Hangzhou, China

**Keywords:** coil, flow diverter, stent, hemodynamics, recurrence

## Abstract

**Objective:**

This study comparatively analyzed the hemodynamic changes resulting from various simulated stent-assisted embolization treatments to explore an optimal treatment strategy for intracranial vertebral artery fusiform aneurysms. An actual vertebral fusiform aneurysm case treated by large coil post-stenting (PLCS) was used as a control.

**Materials and Methods:**

A single case of an intracranial vertebral artery fusiform aneurysm underwent a preoperative and eight postoperative finite element treatment simulations: PLCS [single and dual Low-profile Visualized Intraluminal Support (LVIS)], Jailing technique (single and dual LVIS both simulated twice, Pipeline Embolization Device (PED) with or without large coils (LCs). Qualitative and quantitative assessments were performed to analyze the most common hemodynamic risk factors for recurrence.

**Results:**

Jailing technique and PED-only had a high residual flow volume (RFV) and wall shear stress (WSS) on the large curvature of the blood flow impingement region. Quantitative analysis determined that PLSC and PED had a lower RFV compared to preoperative than did the jailing technique [PED+LC 2.46% < PLCS 1.2 (dual LVIS) 4.75% < PLCS 1.1 (single LVIS) 6.34% < PED 6.58% < Jailing 2.2 12.45% < Jailing 1.2 12.71% < Jailing 1.1 14.28% < Jailing 2.1 16.44%]. The sac-averaged flow velocity treated by PLCS, PED and PED+LC compared to preoperatively was significantly lower than the jailing technique [PED+LC = PLCS 1.2 (dual LVIS) 17.5% < PLCS 1.1 (single LVIS) = PED 27.5% < Jailing 1.2 = Jailing 2.2 32.5% < Jailing 1.1 37.5% < Jailing 2.1 40%]. The sac-averaged WSS for the PLCS 1.2 (dual LVIS) model was lower than the PED+LC, while the high WSS area of the Jailing 1 model was larger than for Jailing 2 [PLCS 1.2 38.94% (dual LVIS) < PED+LC 41% < PLCS 1.1 43.36% (single LVIS) < PED 45.23% < Jailing 2.1 47.49% < Jailing 2.2 47.79% < Jailing 1.1 48.97% < Jailing 1.2 49.85%].

**Conclusions:**

For fusiform aneurysms, post large coil stenting can provide a uniform coil configuration potentially reducing the hemodynamic risk factors of recurrence. Flow diverters also may reduce the recurrence risk, with long-term follow-up required, especially to monitor branch blood flow to prevent postoperative ischemia.

## Introduction

Fusiform aneurysms are more prone to occur in the posterior circulation (1). Intracranial aneurysm recurrence is related to the degree of the parent artery involved (2). Fusiform aneurysms have more extensive wall enhancement than the saccular variety indicating wall inflammation and vulnerability (3, 4). Vulnerable vessel walls exposed to abnormal hemodynamics are susceptible to aneurysm growth, rupture, and recurrence (5–7). For highly involved parent artery aneurysms, coils cannot be safely and effectively used for vascular reconstruction (8). Revascularization therapy mainly relies on various stent-assisted embolization techniques presenting different procedures and recurrence risks (9, 10). Currently, our center's most commonly used stent-assisted embolization techniques include Jailing, post-large-coil stenting (PLCS), and Pipeline Embolization Device (PED) combined with or without large-coil techniques. Jailing techniques with conventional stents present a relatively low procedure-related risk with a high recurrence exposure (11). PLCS proposed in our center can be used to embolize fusiform aneurysms and lower their recurrence rate. Increased off-label use of flow diverters presents some extent ischemic risk for intracranial vertebral artery fusiform aneurysms (12). Unclear hemodynamic effects among the various reconstructive strategies for intracranial vertebral fusiform aneurysms make it difficult to nominate an optimal approach.

Hemodynamically, wall shear stress (WSS) is an important risk factor for aneurysm rupture. Low WSS induces destructive remodeling caused by inflammatory cells, resulting in aneurysm instability. Higher than normal WSS also can result in the enlargement and rupture of aneurysms based on other mechanisms (6). This study focused on the recanalization risk induced by blood inflow for the unruptured aneurysm. To elucidate the issue of postoperative recurrence, high WSS and velocity, larger residual flow volume (RFV), and other hemodynamic characteristics from large blood inflow were correlated with recanalization and recurrence (13–18). Luo et al. (16) reported high WSS and flow velocity in partially occluded saccular aneurysms prone to recanalization. Umeda et al. (18) found that RFV predicts the recurrence of coiled paraclinoid aneurysms. For large narrow-necked aneurysms, PED with coils treatment can accelerate thrombotic efficiency, favoring aneurysm occlusion in the competition with delayed rupture (19).

However, no CFD mechanism-related studies exist on the potential recurrence risk among different reconstruction techniques for intracranial vertebral artery fusiform aneurysms. This present study modeled an actual case of vertebral artery fusiform aneurysm treated with PLCS without considering thrombosis, simulating and comparing the preoperative hemodynamic effects and eight post-operative finite element treatment simulations– PLCS x 2 (single and dual Low-profile Visualized Intraluminal Support (LVIS)), Jailing technique x 4 (single and double LVIS both simulated twice with different coil configurations), and 2x Pipeline Embolization Device (PED) with or without large coils (LCs) – to analyze the most common hemodynamic risk factors for recurrence.

## Materials and Methods

### Population

**Figure 1:**
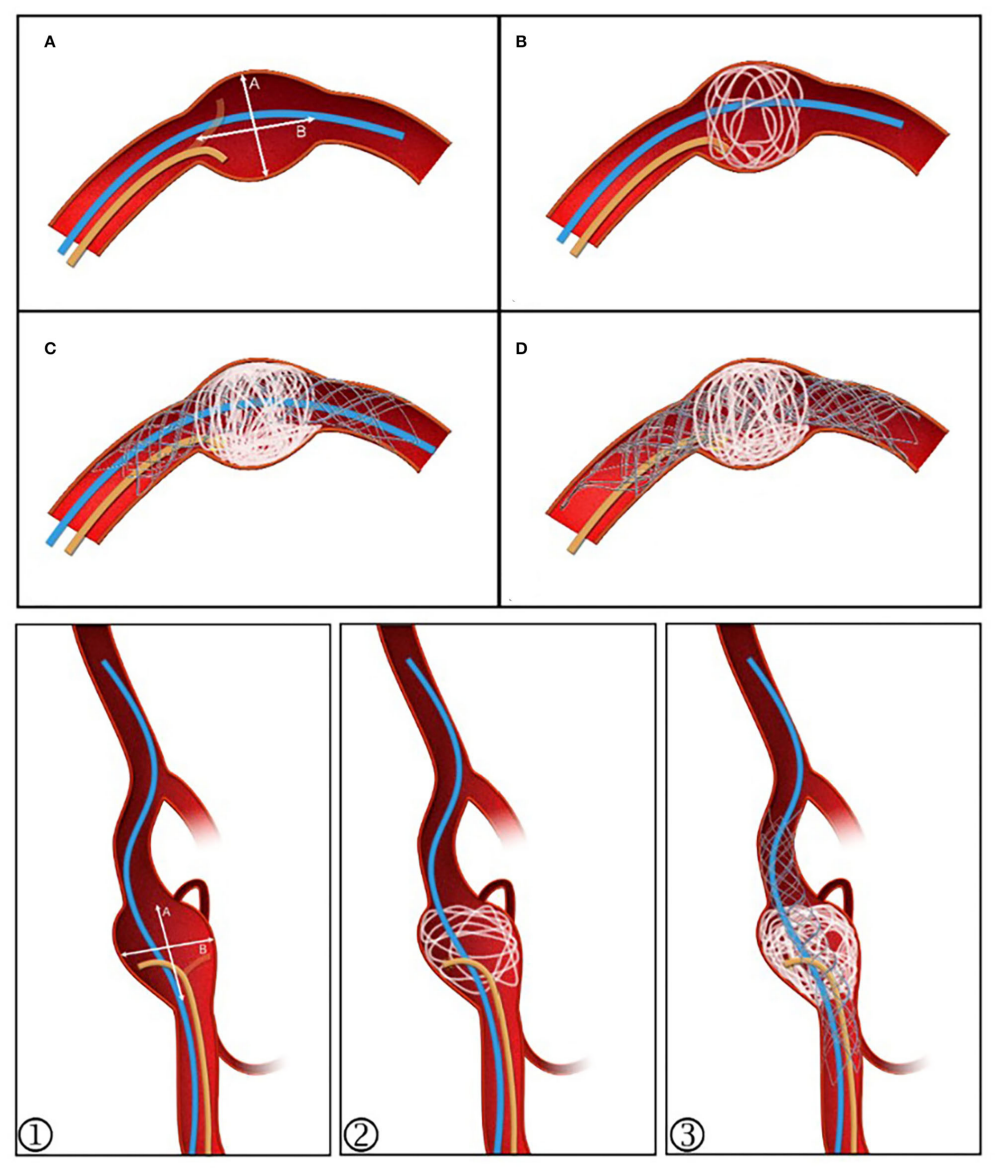
Post-large coil stenting technique schematic diagram. **(A)** The stenting microcatheter (blue) and unshaped coiling microcatheter (yellow) are positioned. **(B)** Coil diameter is selected with reference to the value ≥A. The aneurysm sac is evenly filled. **(C)** Continued embolization using 2–4 coils, then deploying the stent and placing the stenting microcatheter at the distal segment as a backup. **(D)** If the sac is not densely embolized or coil protrusion into the stent occurs, a second stent can be released to provide flow diversion, allowing further embolization to proceed. ①–③ For those with branches or an irregular sac, the stent can be semi-released to assist in forming a basket while protecting the branches.

**Figure 2:**
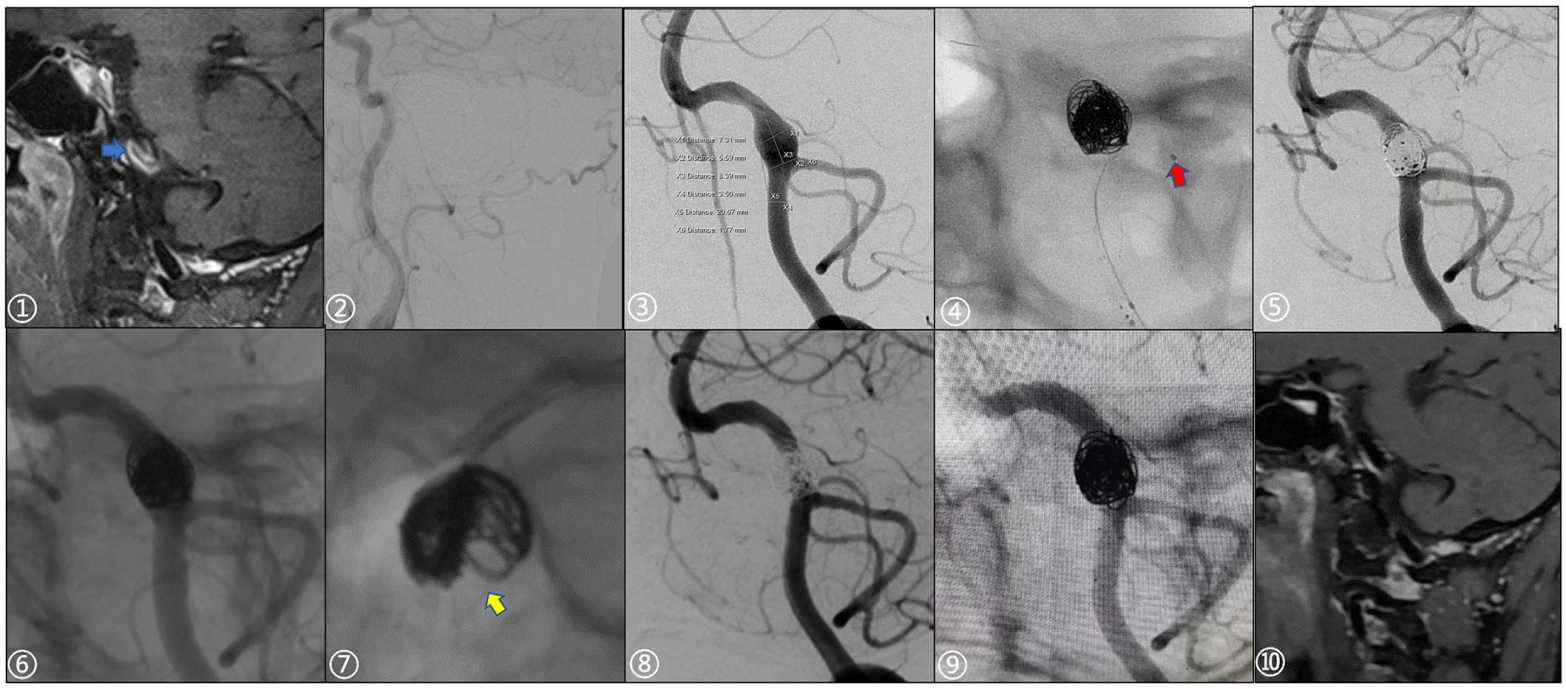
A 49-year-old male patient presented with a sudden severe headache once 2 months prior. ① Preoperative high-resolution MRI showed significant enhancement of the vessel wall with the intraluminal slow flow (blue arrow). ② Right vertebral artery dysplasia. ③ Preoperative measurement of aneurysm and parent artery, aneurysm size: 7.31 x 8.39 mm. ④ Echelon-10 microcatheter (red arrow) was used to protect the posterior inferior cerebellar artery during operation. ⑤, ⑥ Stents were post-deployed, Immediate postoperative angiography and non-subtraction images showed slight stagnation in the aneurysm sac (Raymond IIIa; Microplex-10 coils: two 8 x 30 cm and one 7 x 30 cm; LVIS stents: 4.5 x 20 mm, 4.5 x 15 mm). ⑦ The distribution of the coils along the wall was not well-uniform. ⑧, ⑨ 12-month follow-up showed that there was no recurrence of the aneurysm (Raymond I) and the parent artery was patent. ⑩ 12-month follow-up with HR-MRI, the vortex in the sac disappeared, while the aneurysm wall was still partially enhanced.

A man in his 40 s with an intracranial fusiform aneurysm in the dominant vertebral artery experienced a sudden headache once 2 months ago. The left vertebral artery fusiform aneurysm diagnosed on MRI in a local hospital was treated with PLCS techniques (schematic Figure 1 for details). Three large coils compared to aneurysmal width (two Microplex-10 8 mm x 30 cm and one 7 mm x 30 cm) plus two LVIS stents (4.5 x 20 mm and 4.5 x 15 mm) were implanted with a modified Raymond IIIa outcome (Figure 2). The 12-month DSA follow-up showed no recurrence or remnant (Raymond I).

### Model Reconstruction

**Figure 3:**
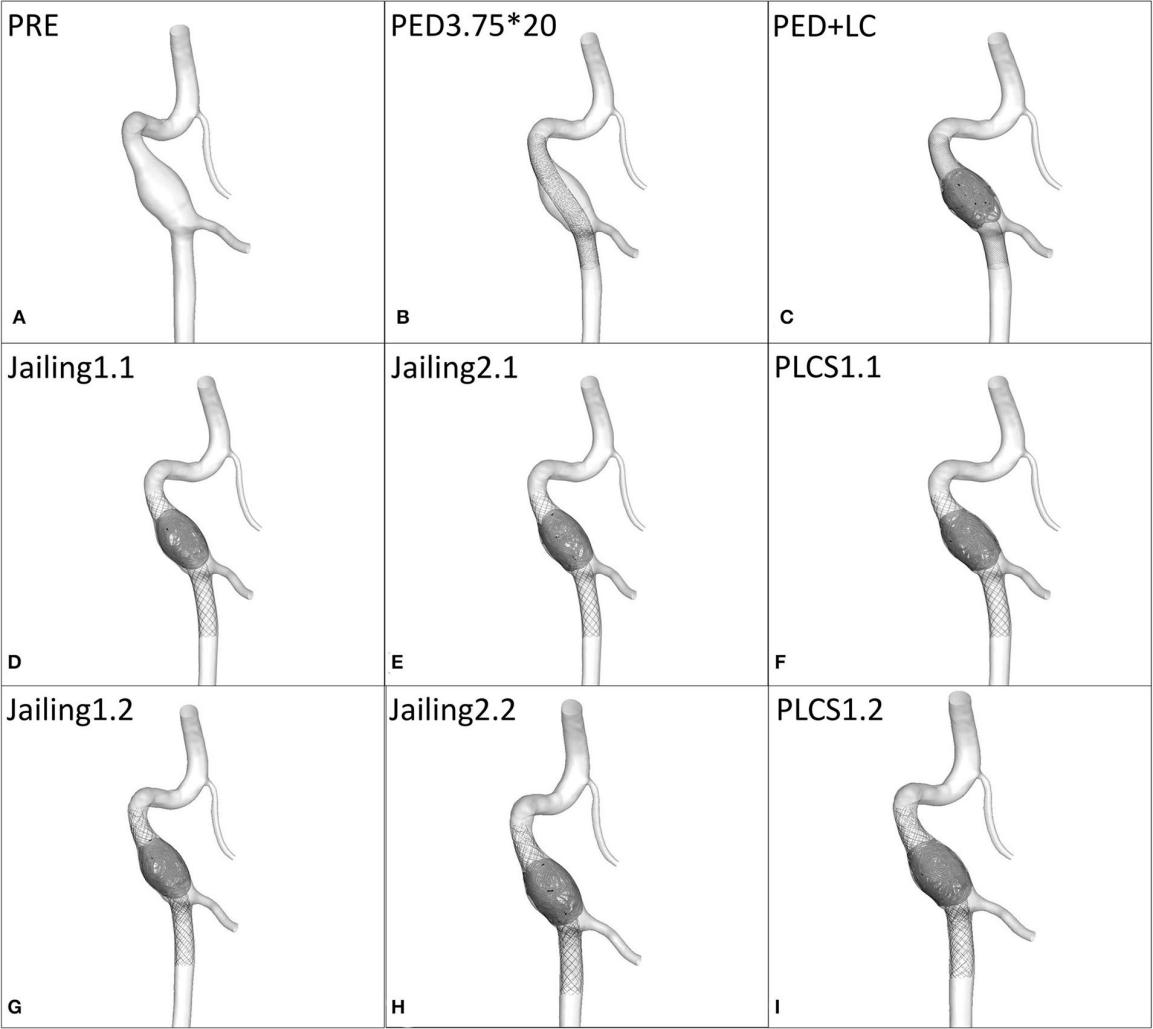
Simulation reconstruction model of aneurysm preoperatively and for eight different treatment approaches. **(A)** Preoperative aneurysm model. **(B)** Pipeline device (PED) 3.75*20 mm implantation model. **(C)** Large coil; Microplex-10 8 x 30 cm x 2 + 7 x 30 cm and PED-3.75 x 20 mm. **(D–F)** Single LVIS stent 4.5 x 20 mm (Jailing 1.1, Jailing 2.1, and PLCS 1.1). **(G–I)** Dual LVIS stent 4.5 x 20 mm, 4.5 x 15 mm (Jailing 1.2, Jailing 2.2, and PLCS 1.2).

Raw data was generated from DSA rotational angiography (high-pressure injector rate 3 ml/s, time 5 s, total volume 15 ml) using Siemens equipment Axiom Artis Zeego, Siemens Medical Solutions, Erlangen, Germany). The acquired raw data were reconstructed in Mimics 17 software (Materialise, Leuven, Vlaams-Brabant, Belgium) to generate STL files subsequently imported into Geomagic 12 software for model repair, trimming, and smoothing (Figure 3A).

### Finite Element Simulation

A two-step finite element simulation of stent deployment was devised (20). Firstly, LVIS and Pipeline models were generated in SolidWorks (Dassault Systems, SolidWorks Corp., MA) according to geometric information (21). Secondly, stent deployment was simulated in ABAQUS v6.14 (SIMULIA, Providence, RI) using the Dynamic Explicit Method and B31 element type, which was also done for the coils. Stent-specific parameters obtained from a previous study (22) were divided into three steps: compression, delivery, and release of the stent. Initially, the stent in its fully released state was inserted into a round tube and compressed to a state where it can be inserted into the micro-catheter model by allocating the displacement load of the outer wall. Then, the stent followed the delivery path of the micro-catheter by providing the displacement load to be delivered to the target area. Finally, the micro-catheter was withdrawn, and the stent was released using a predefined stress-strain field allowing the stent to expand in the designated area to fit the inner wall of the artery. The delivery path was generated by connecting the center points of the blood vessel cross-section, while the stent release point was determined by the surgical image. The “general contact” algorithm was used in ABAQUS to deal with the complex interactions during stent release, with the friction coefficient assigned to 0.15 (23).

The simulation of the coil insertion process was carried out in ABAQUS (20). The process involved both pulling in and pushing out the coil. The coils were generated in MATLAB (MathWorks, Natwick, MA) using centerlines to simplify the coil shape (22). First, a coil-microcatheter-aneurysm model was built using NX12.0, and then the model was imported into ABAQUS. The coil is pulled into the microcatheter by distributing a displacement load at one end of the coil, while the coil inside the microcatheter is pushed out into the aneurysm using a pre-defined stress-strain field via creating a displacement load on the other end of the coil. Finally, the coil was placed in the aneurysm sac and then scanned in three dimensions according to the centerlines after placement (24, 25). The coil exhibited the following physical properties: a density of 2.13 × 10^−8^kg/m^3^, Young's modulus of 10,000 Pa, and Poisson's ratio of 0.39 (26). The three-dimensional stent and coil models obtained by finite element simulation were output as STL format files, maintaining the same spatial coordinate system as the blood vessel model in the next step of the hemodynamic simulation.

The simulated hemodynamics of the preoperative untreated model was adopted as baseline parameters. The jailing technique with coiling was conducted twice, to simulate both separated and connected coils. A total of eight postoperative treatment options were simulated (Figure 3): 1. PED implantation; 2. PED + large coils; 3–6. Jailing technique with two coiling simulations (1.1 single/1.2 dual LVIS with separated coils and 2.1 single/2.2 dual LVIS with connected coils); 7–8. For post-large-coil stenting (PLCS1.1 single/1.2 dual LVIS). Three large-coils (two Microplex-10 8 mm x 30 cm and one 7 mm x30 cm) and two stents (LVIS 4.5 mm x 20 mm and 4.5 mm x 15 mm) were selected. The PED size (3.75^*^20 mm) was determined by two neuro-interventionists with over 10-year experience.

### Hemodynamic Simulation

The virtual treatment model is subjected to CFD simulation analysis. To generate mesh files, the preoperative and eight postoperative models were imported into ANSYS ICEM CFD version 16.2 (ANSYS Inc, Canonsburg, PA, USA). A grid independence test was performed to determine the appropriate grid size for the stability of the calculation outcomes and the efficiency calculation. Due to the different geometric dimensions of vessels, stents, and coils, the mesh sizes of different object surfaces are determined to various values. The grid size for the stent wire surface was finally set to 1/6 of the circumference of the wire. The artery and coil surface were 0.16 mm along with the 0.03 mm LVIS surface and the 0.015 mm PED surface grid. A three-layer boundary mesh was added to improve the accuracy of the simulation results in the near-surface region of the model. Final mesh calculations were generated as follows: 3 million for the preoperative model; 96 million for PED and PED with large coil; 45 million for Jailing1.1, 1.2, and PLCS1.1; 65 million for Jailing 2.1, 2.2, and PLCS1.2. The hemodynamic simulations were fitted with the Navier-Stokes equations for steady-state simulations using ANSYS CFX version 2019 (ANSYS Inc, Canonsburg, PA, USA). The blood was designed as an incompressible, laminar flow, Newtonian fluid with a density of 1,056 kg/m^3^ and a viscosity of.0035 kg/m·s (27). The vessel wall was designed to be rigid with no slip. The flow rate for the vertebral artery inlet was set at 1.3 ml/s (28). Outlet conditions were calculated according to Murray's law of flow distribution (29). A steady coupled solver was used for laminar simulation. The residual target of the convergence criterion was 0.00001.

### Statistical Analysis of the Various Approaches

Qualitative and quantitative methods were used to analyze and compare the flow velocity, WSS, and RFV (v >0.03 m/s) (19) in the aneurysm sac among nine simulations (preoperative and eight postoperative simulations). The vascular segment covered by the stents was intercepted and the aneurysm sac volume was defined as the space between the vascular wall and the stent surface. Defining the preoperative hemodynamic parameters as 100%, the hemodynamic changes for each of the treatment strategies were analyzed and compared.

## Results

### Residual Blood Flow Volume (RFV)

#### Qualitative Analysis

**Figure 4:**
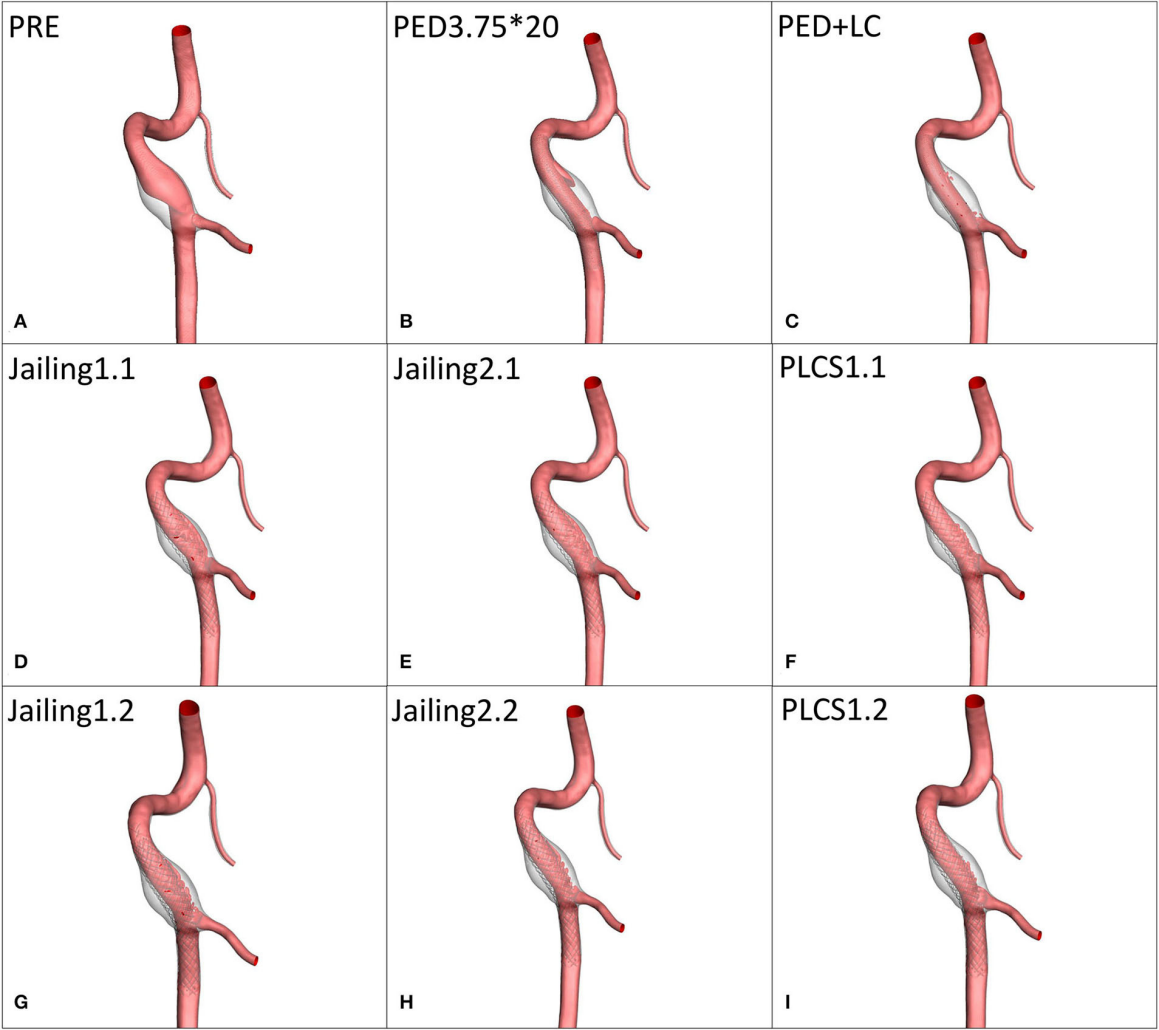
**(A)** Preoperative high-velocity region (v > 0.03 m/s). **(B–I)** RFV maps for different stent-assisted techniques.

**Figure 5:**
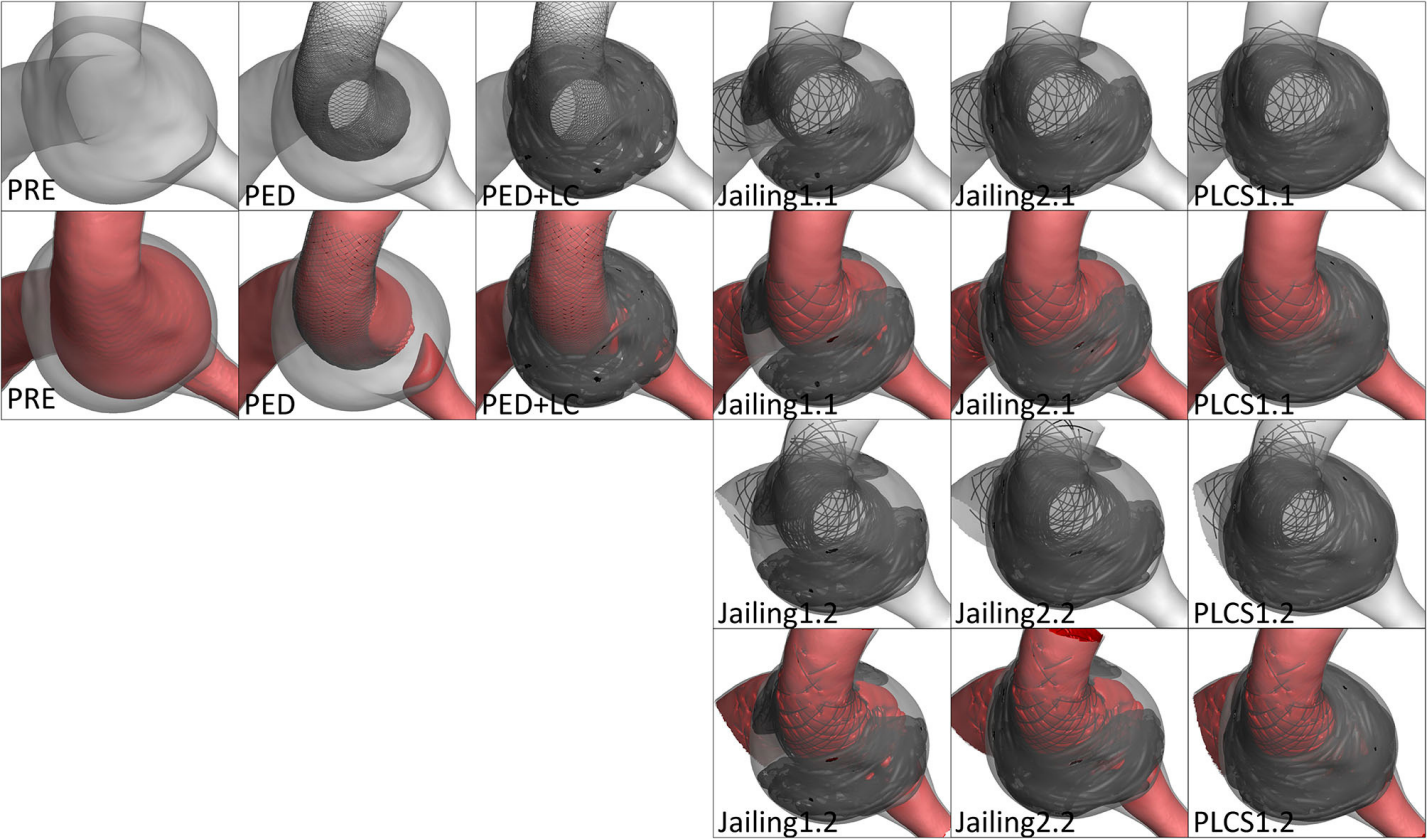
The down-the-barrel view of preoperative and different stent-assisted technique simulation treatment.

RFV was discerned in the sac after PED implantation alone. According to the simulated projection and down-the-barrel view, the Jailing technique had a higher RFV on the large curved side due to the non-uniformity of the coil embolization, more similar to PED-only implantation than the RFV values of the PED+LC and PLCS techniques (Figures 4, 5).

#### Quantitative Analysis

**Figure 6:**
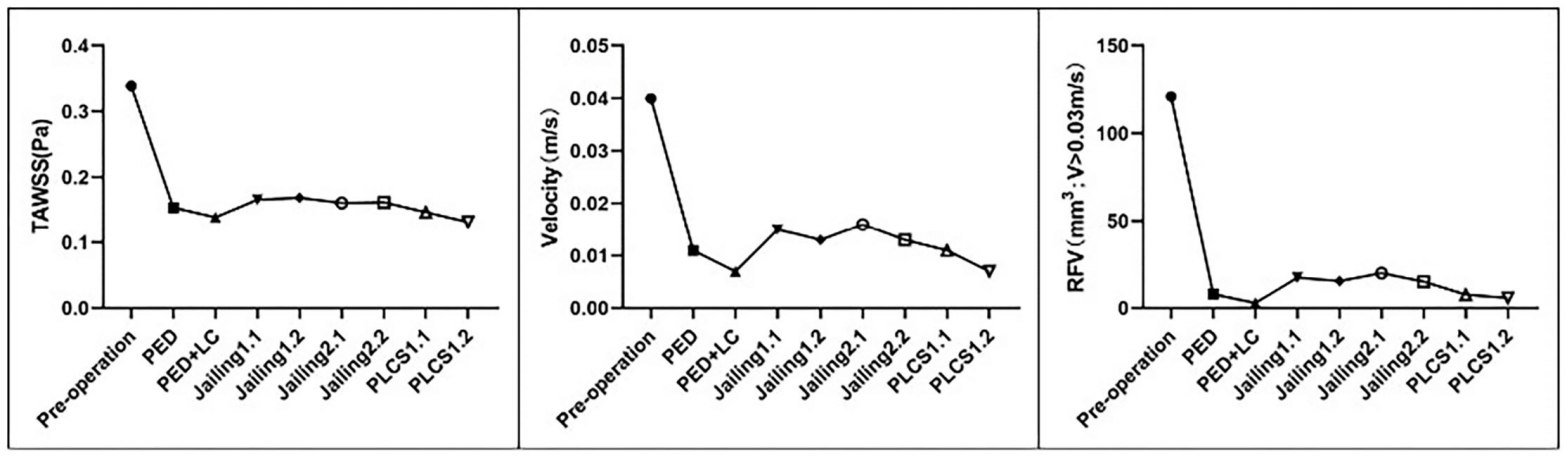
Quantitative assessment of the sac-averaged WSS, sac-averaged velocity, and RFV of the simulated preoperative and various stent-assisted models.

Both PLCS and PED with or without large-coils had lower residual percentages of RFV than the Jailing technique compared with pre-operation. The residual percentage of RFV of for dual stents in PLCS and Jailing are smaller than for the single stent [PED + LC 2.46% < PLCS 1.2 (dual LVIS) 4.75% < PLCS 1.1 (single LVIS) 6.34% < PED 6.58% < Jailing 2.2 12.45% < Jailing 1.2 12.71% < Jailing 1.1 14.28 % < Jailing 2.1 16.44%; Table 1, Figure 6].

**Table 1:** Hemodynamic parameters pre-and post-operative stimulation treatment for various stent-assisted techniques.

**Parameters**	**Pre**	**PED**	**PED+LC**	**Jailing1.1**	**Jailing1.2**	**Jailing2.1**	**Jailing2.2**	**PLCS1.1**	**PLCS1.2**
Sac-averaged WSS (Pa)	0.339	0.154	0.139	0.166	0.169	0.161	0.162	0.147	0.132
Sac-averaged velocity (m/s)	0.040	0.011	0.007	0.015	0.013	0.016	0.013	0.011	0.007
RFV (mm^3^; v > 0.03m/s)	121.12	7.964	2.975	17.300	15.397	19.907	15.074	7.678	5.753

### The Average Flow Velocity of the Aneurysm Sac

**Figure 7:**
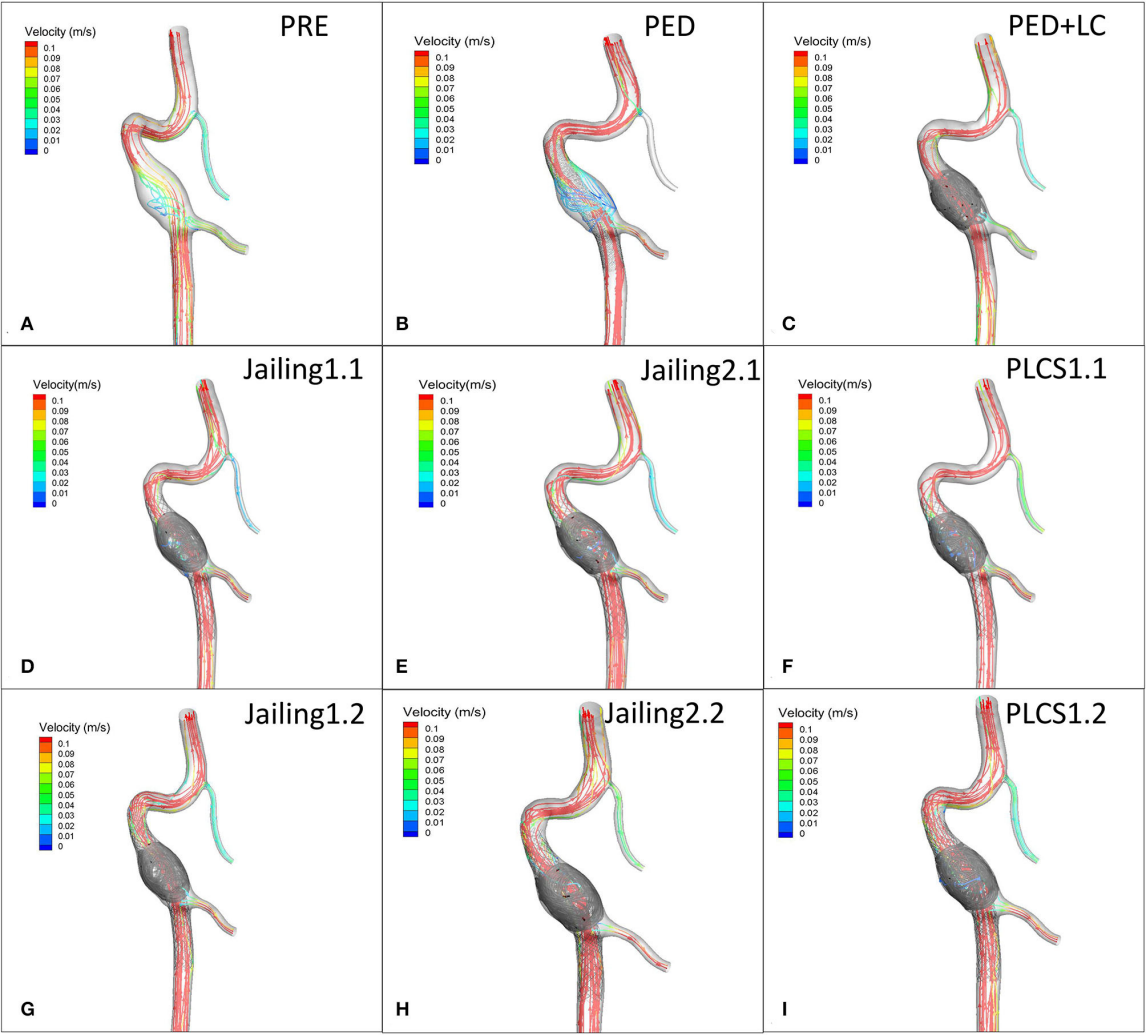
**(A)** Preoperative velocity streamlines. **(B–I)** Post-operative velocity streamlines for PED, PED+LC, Jailing, and PLCS models.

The streamline diagram and quantitative analysis showed that the averaged flow velocity in the aneurysm sac after PLCS for PED with and without large-coils decreased significantly more than for the Jailing technique. The sac-averaged flow velocity for dual stents in PLCS and Jailing technique are smaller than single stent [PED+LC = PLCS1.2 (double LVIS) 17.5% < PLCS1.1 (single LVIS) = PED 27.5% < Jailing1.2 = Jailing2.2 32.5% < Jailing1.1 37.5% < Jailing2.1 40%; Table 1, Figures 6, 7].

### Average WSS of the Aneurysm Wall

#### Qualitative Analysis

**Figure 8:**
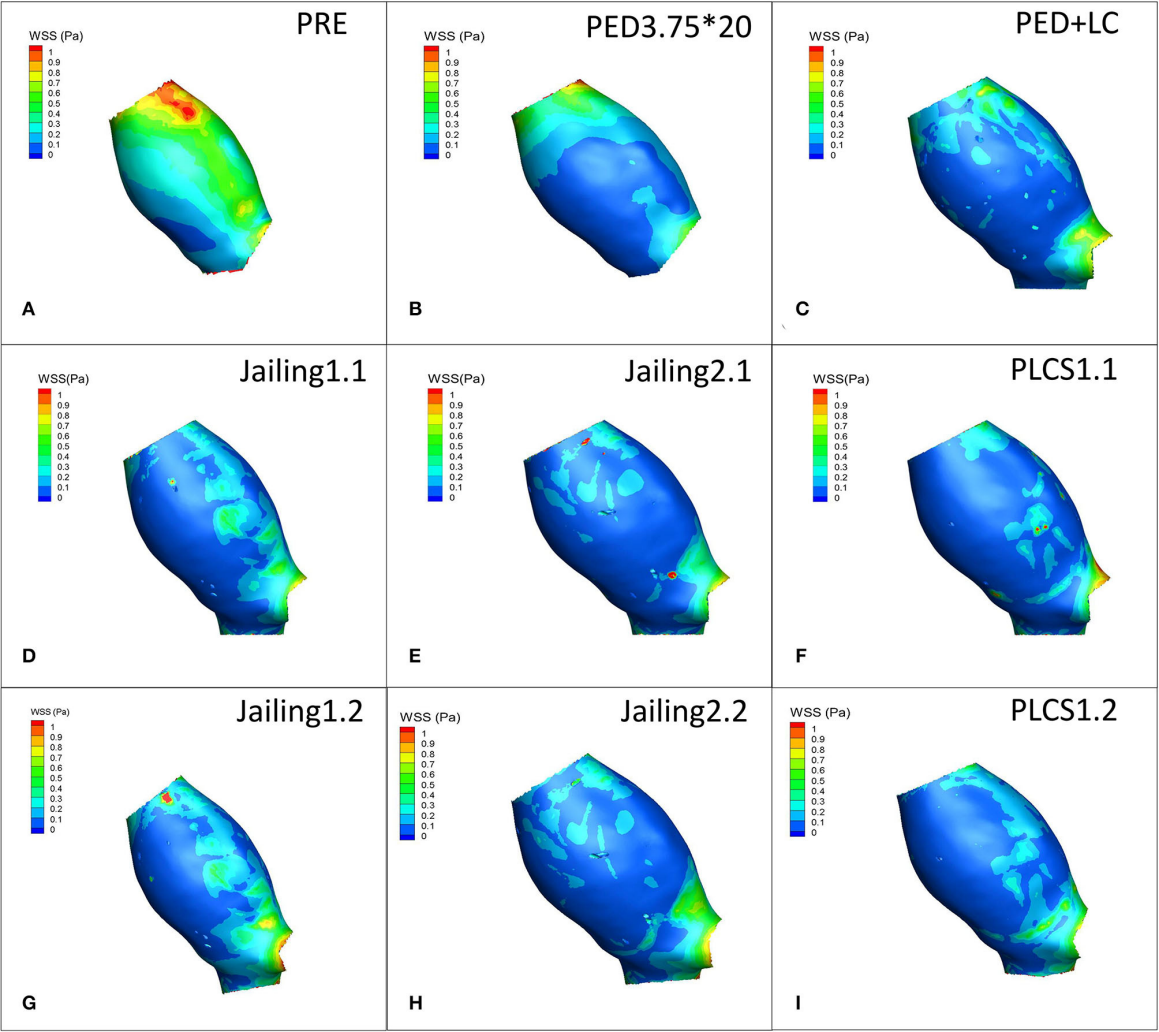
**(A)** Preoperative WSS. **(B–I)** WSS of PED, PED+LC, Jailing, and PLCS post-operative models.

WSS values of all postoperative models decreased significantly compared with pre-operation. Jailing 1 experienced a larger high WSS region than Jailing 2 (Figure 8).

#### Quantitative Analysis

WSS from the PLCS 1.2 (double LVIS) had the largest decline [PLCS1.2 (Dual LVIS) 38.94% < PED+LC 41% < PLCS1.1 (Single LVIS) 43.36% < PED 45.23% < Jailing2.1 47.49% < Jailing 2.2 47.79% < Jailing 1.1 48.97% < Jailing1.2 49.85 %; Table 1, Figures 6, 8].

## Discussion

The main conclusion of this study is that PLCS and PED with or without large-coils can significantly decrease the hemodynamic risk factors of recurrence in the treatment of fusiform vertebral aneurysm compared with the jailing technique. Hemodynamic studies have shown that high WSS, large RFV, and high-velocity areas after interventional treatment are risk factors for aneurysmal recurrence (17, 19, 30). Chatziprodromou et al. and Rayz et al. (31, 32) reported that high blood flow velocity and high WSS are often accompanied, which is not conducive to thrombosis in the aneurysm sac and has an adverse impact on the long-term stability after embolization. Hemodynamic risk parameters – WSS, RFV, and high-velocity regions – were lower for PLCS than those for jailing, thus reducing the recurrence risk. In both jailing and PLCS technique, the hemodynamic risk factors of the single LVIS stent were larger than those for dual LVIS stents. Therefore, the overlapping stent technique is a beneficial option to reduce the potential risk of recurrence compared with a single stent. At present, few studies analyze the recurrence of vertebral artery aneurysms based on CFD (33, 34). These studies indicated that hemodynamics played a role in vertebral fusiform aneurysms similar to saccular aneurysms. Although the data, in this case, are based on the vertebral artery, the results should be generalizable to other aneurysms with similar morphological characteristics.

Quantitative analysis showed hemodynamic risk factors for recurrence after jailing was also confirmed higher than PLCS and PED techniques due to incomplete embolization from the micro-catheter fixed by the stent (35). Hong et al. (36) proposed that the semi-Jailing technique could help improve the maneuverability of the micro-catheter during the treatment of wide-necked complex aneurysms. Chen et al. (10) advocated a modified balloon-in-stent technique for the treatment of fusiform aneurysms. Prolonged balloon inflation and stent malposition potentially lead to thromboembolic events. The wide extent of inflammation on the fusiform aneurysm wall indicated vulnerability to recurrence (3, 37). The uniform distribution of coils helps create a local flow diversion effect which can reduce recurrence. The inflow tract WSS for Jailing 1 is larger than Jailing 2 mainly due to a failure to fully pack the impingement area, consistent with the recurrence of saccular aneurysms after embolization (38, 39). This suggests that more emphasis should be placed on the inflow tract.

Procedure critical points of the PLCS technique on coil, stent, and embolizing microcatheter selection should be emphasized for fusiform aneurysms. Coil packing has some inherent randomness in clinical practice. Coil configuration for PLCS should achieve a more even distribution than the jailing technique. The framing coils (diameter ≥ aneurysm width) often require repeated adjustment contributing to uniform coil distribution. While protecting the patency of the involved branches, semi-deployment of the stent can assist in framing the large coils. Soft coils with a smaller primary helix diameter were preferred to result in stent well-apposition. The proper amount of large-coils (2–4) is normally used to prevent stent opening failure. Small coils with a rivet technique may result in stenosis or delayed occlusion of the parent artery. LVIS stents were more usually selected for the PLCS technique due to a number of advantages. LVIS stents can be re-sheathed and pushed during the deployment process (9) allowing for the uniform distribution of large coils. Further, LVIS stent is tied to lower thrombogenicity than Pipeline stents. Finally, LVIS stents are more cost-effective than Pipelines (10). However, due to the local dense coverage rate, antiplatelets should be rigorously confirmed to avoid any delayed vessel occlusion. Previous studies have reported a wide range of LVIS in-stent stenosis rates between 17.5 and 86.7% (40, 41). According to previous studies, multiple flow diverters without coils were not recommended for ruptured lesions due to the necessity of strict antiplatelet therapy (42). On the other hand, treatment with overlapping PEDs also presents a high ischemic risk (12), such that non-overlapping stents might be preferable. Multiple flow diverters were not simulated in this present study. Using a Pipeline device with large coils is a promising technique for fusiform aneurysms, especially for aneurysms without evident perforators or branches. An unshaped or 45-degree tip can provide sway to the micro-catheter permitting greater maneuverability and thus the creation of more uniform baskets. For such patients, antiplatelet medication should be administered cautiously in the peri- and postoperative phase, with timely adjustment according to TEG and CYC2P19 gene results.

### Limitations

This study has the following limitations. This proof-of-concept study was a single case. However, it effectively demonstrates hemodynamic effects after treatment for different stent-assisted techniques. Comprehensive intracranial stents such as Solitaire and Neuroform were not tested. In addition, not all treatment strategy combinations were simulated such as Jailing + Pipeline. Since a Murray flow outlet was employed in this study, the flow rate change of the PICA branch was inapplicable. The pressure outlet should be a feasible way to evaluate the influence on the flow rate of PICA. Flow change may be affected by thrombus and stent endothelialization. This study did not address the degree of vascular curvature which can affect the different stent-assisted strategies. The coil distribution from the PLCS technique is somewhat random and the simulation cannot be completely consistent with actual placement. This CFD study used common assumptions such as rigid walls and a lack of specific inflow and outflow tract flow conditions.

## Conclusions

For fusiform aneurysms of the intracranial vertebral artery, the PLCS technique can more uniformly pack aneurysm sacs and may reduce the hemodynamic risk factors of recurrence similar to flow diverters, though long-term follow-up is still required. Attention should be paid to the impact on any perforators or branches to lessen the ischemic risk.

## Data Availability Statement

The raw data supporting the conclusions of this article will be made available by the authors, without undue reservation.

## Ethics Statement

The studies involving human participants were reviewed and approved by the Institution Review Board of Huashan Hospital affiliated to Fudan University approved this retrospective study and waived the requirement for informed consent. Written informed consent for participation was not required for this study in accordance with the national legislation and the institutional requirements.

## Author Contributions

XZ and JX conceived and designed the research and handled the funding and supervision. YJ acquired the data and drafted the manuscript. YJ, GLu, LG, GLi, and RZ analyzed and interpreted the data. YJ, XL, and HW performed the statistical analysis. All authors made critical revisions to the manuscript for important intellectual content and reviewed the final version of the manuscript.

## Funding

This work was partially supported by the National Natural Science Foundation of China (No. 81771242).

## Conflict of Interest

GLi, RZ, XL, and JX were employed by the company ArteryFlow Technology Co., Ltd. The remaining authors declare that the research was conducted in the absence of any commercial or financial relationships that could be construed as a potential conflict of interest.

## Publisher's Note

All claims expressed in this article are solely those of the authors and do not necessarily represent those of their affiliated organizations, or those of the publisher, the editors and the reviewers. Any product that may be evaluated in this article, or claim that may be made by its manufacturer, is not guaranteed or endorsed by the publisher.
